# I-HEDGE: determining the optimum complementary sets of taxa for conservation using evolutionary isolation

**DOI:** 10.7717/peerj.2350

**Published:** 2016-08-23

**Authors:** Evelyn L. Jensen, Arne Ø. Mooers, Adalgisa Caccone, Michael A. Russello

**Affiliations:** 1 Department of Biology, University of British Columbia, Okanagan Campus, Kelowna, BC, Canada; 2 Biological Sciences, Simon Fraser University, Burnaby, BC, Canada; 3 Department of Ecology and Evolutionary Biology, Yale University, New Haven, CT, United States

**Keywords:** Conservation genetics, HEDGE, Mitochondrial control region, Shapley index, Noah’s Ark problem

## Abstract

In the midst of the current biodiversity crisis, conservation efforts might profitably be directed towards ensuring that extinctions do not result in inordinate losses of evolutionary history. Numerous methods have been developed to evaluate the importance of species based on their contribution to total phylogenetic diversity on trees and networks, but existing methods fail to take complementarity into account, and thus cannot identify the best order or subset of taxa to protect. Here, we develop a novel iterative calculation of the heightened evolutionary distinctiveness and globally endangered metric (I-HEDGE) that produces the optimal ranked list for conservation prioritization, taking into account complementarity and based on both phylogenetic diversity and extinction probability. We applied this metric to a phylogenetic network based on mitochondrial control region data from extant and recently extinct giant Galápagos tortoises, a highly endangered group of closely related species. We found that the restoration of two extinct species (a project currently underway) will contribute the greatest gain in phylogenetic diversity, and present an ordered list of rankings that is the optimum complementarity set for conservation prioritization.

## Introduction

The Noah’s Ark problem embodies the difficulties of deciding what to conserve in the face of limited resources (Weitzman, 1998). It is generally recognized that the extinction of some species represents a greater loss of biodiversity than others (an example is the extinction of one among many species of rat versus extinction of the panda, see Vane-Wright, Humphries & Williams, 1991). In the midst of the current biodiversity crisis, if prioritization is required, conservation efforts should be directed towards ensuring that extinctions do not result in inordinate losses of evolutionary history (Vane-Wright, Humphries & Williams, 1991). Methods first pioneered by Faith (1992) have been further developed and refined to evaluate the relative importance of species based on their contribution to total genetic diversity (Weitzman, 1992; Witting & Loeschcke, 1993; Redding, 2003; Steel, Mimoto & Mooers, 2007; Faith, 2008; Haake, Kashiwada & Su, 2008; Minh, Klaere & Von Haeseler, 2009; Hartmann, 2013). These methods were initially created for the analyses of phylogenetic trees, but have recently been extended for use with phylogenetic networks that better represent genetic diversity among populations and recently diverged species (Volkmann et al., 2014).

Current metrics consider the expected contribution of each taxon to future subsets of taxa (i.e., scenarios where some taxa are lost). One is the “fair proportion” or “evolutionary distinctness” metric (Redding, 2003; Isaac et al., 2007; Jetz et al., 2014) extended to networks, where all future subset sizes and identities are considered equally-likely (referred to here as the Shapley index, SH, following Haake, Kashiwada & Su, 2008). Another, heightened evolutionary distinctiveness (HED), explicitly weighs future subsets by their probability using estimates of the current extinction probabilities of all other taxa (Steel, Mimoto & Mooers, 2007).

Rankings based on these metrics alone do not necessarily constitute rational prioritizations for conservation. One issue is that a secure species on a long branch may have a high HED score, because its own low probability of extinction [p(ext)] does not contribute to its own score. Also, as laid out clearly by Faith (2008), the above metrics are not designed to identify the best ordering or *subset* of taxa to protect, since complementarity is not taken into account. For example, two closely related species may both be at high risk of extinction, meaning each would contribute to future diversity if its relative were to go extinct. However, if one of the two were successfully protected, its sister should drop in value because the shared component of diversity is now retained.

The first issue above has been addressed by the development of metrics such as HEDGE (“heightened evolutionary distinctiveness and globally endangered,” Steel, Mimoto & Mooers, 2007), which is the product of a taxon’s HED score and p(ext). HEDGE scores represent the increase in expected phylogenetic diversity if the taxon’s p(ext) is changed from its current value to a p(ext) of zero (i.e., it is “saved” from extinction; see also Faith, 2008). Here, we present an extension of HEDGE that addresses the issue of complementarity. If the species that has the highest HEDGE score is indeed saved from extinction, then the HED score of neighbouring taxa should decrease to reflect this new p(ext) of the shared part of the network. We propose a modified, iteratively calculated, version of HEDGE (I-HEDGE), which is calculated by “saving” the top ranked taxon after calculating HEDGE in each round by setting its extinction probability to near zero, and then recalculating HEDGE until all species have been “saved.” This procedure produces the optimal ranked list for conservation prioritization, taking into account complementarity and based on both phylogenetic diversity and extinction probability.

**Figure 1:**
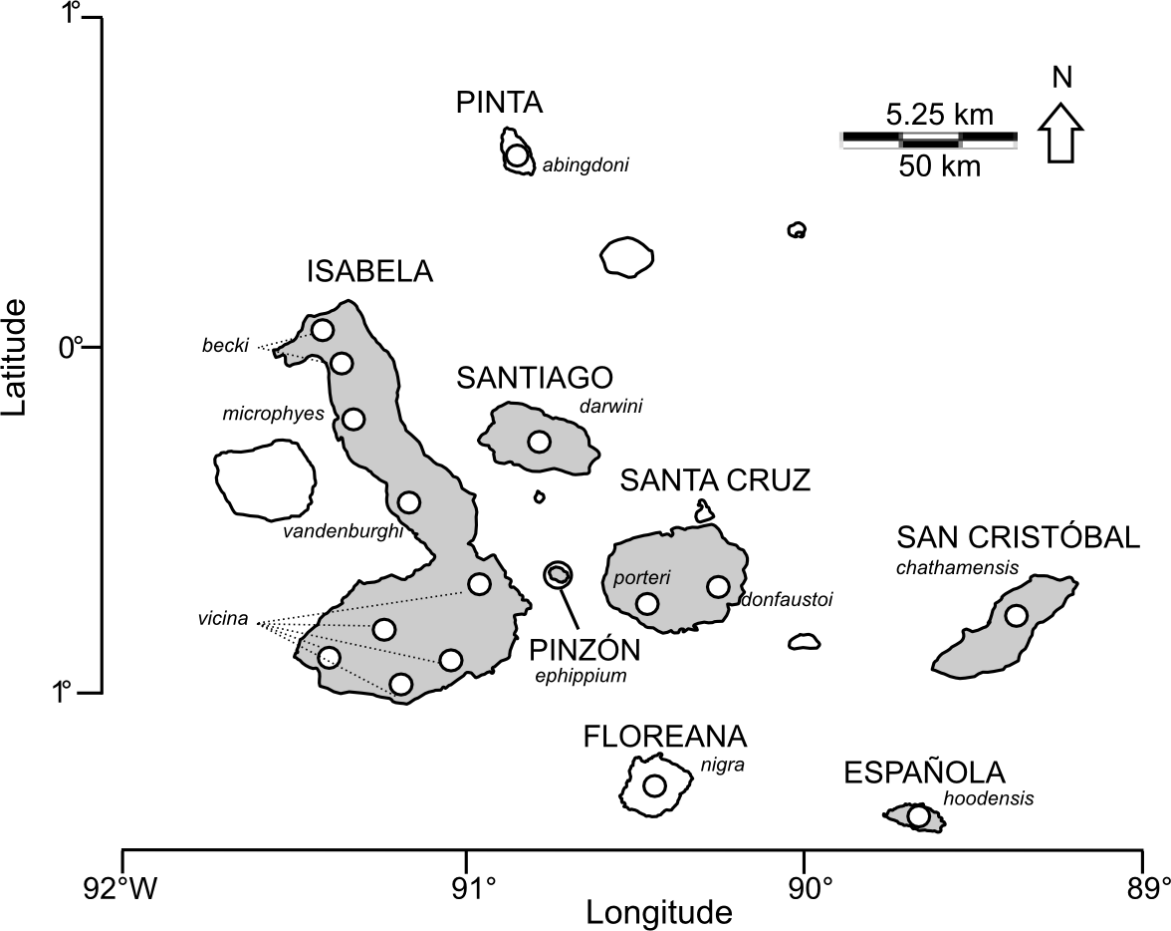
Map of the Galápagos archipelago showing locations of *Chelonoidis* tortoise populations. Names of islands are in capital letters; species epithets are indicated in italics. Circles indicate locations for giant tortoise populations. Islands shaded in grey have extant populations of giant tortoises.

To demonstrate this procedure, we use the example of the giant Galápagos tortoises (genus *Chelonoidis*), a recent island radiation with complex phylogeography and hierarchical levels of divergence (Fig. 1). Recently diverged groups, such as island radiations, are the type of system where a network-based ranking approach will be most relevant. Tortoises initially colonized Galápagos approximately 3 million years ago from mainland South America, and subsequently radiated across all major islands and volcanoes as they formed (Caccone et al., 2002; Poulakakis et al., 2012). Historically, 15 species were formally described and were abundantly distributed across the Galápagos archipelago (MacFarland, Villa & Toro, 1974), exhibiting divergence times spanning a wide-temporal range (<0.28 mya—1.7 mya; Caccone et al., 2002; Poulakakis et al., 2012). Populations were decimated throughout the 18th-20th centuries through human exploitation and the negative impacts of invasive species. Four species have gone extinct, and several others have been taken to the brink (MacFarland, Villa & Toro, 1974). Over the past 50 years, conservation efforts have been extensive, targeted primarily at the most imperilled species. Although effective at preventing the extinction of two additional species and increasing population sizes of others, these conservation strategies have been designed and implemented without reference to the level of genetic divergence and distinctiveness of individual populations, raising concerns that this approach may not maximize genetic diversity in the future.

Here, we present I-HEDGE, a procedure to determine the optimum complementarity set for conservation prioritization, and explore its utility in the network-based context of ranking the giant Galápagos tortoise species. We compare the I-HEDGE approach to the Shapley index, a simpler, non-complementarity method on networks which is directly equal (Volkmann et al., 2014) to the Fair Proportion metric used by the Zoological Society of London in their Edge of Existence program (Isaac et al., 2007). The results are discussed in light of past and current conservation strategies directed towards giant Galápagos tortoises.

## Materials and Methods

### Data set

Previous studies of giant Galápagos tortoises have resulted in the development of a database of mitochondrial control region (CR) sequence data from population-level samples of all extant and several extinct species (Caccone et al., 2002; Russello et al., 2005; Russello et al., 2007; Poulakakis et al., 2008; Garrick et al., 2012; Poulakakis et al., 2012; Edwards et al., 2013). Here, we made use of that database (DRYAD entry doi: 10.5061/dryad.7h8q2), consisting of 334 individuals from extant species sampled across 15 sites, in addition to 33 individuals from two extinct species (Russello et al., 2007; Poulakakis et al., 2008; Russello et al., 2010; Garrick et al., 2012; Edwards et al., 2013) (see Table 1). We have included the extinct species in our study because they may not be extinct for much longer, as the Galápagos National Park has initiated a program to rebreed them from living individuals with admixed ancestry (see Discussion). Here, we have performed the analyses using the currently accepted taxonomy, including recognizing the recently described species, *C. donfaustoi* (Poulakakis et al., 2015), as distinct from *C. porteri* on Santa Cruz Island.

**Table 1:** Sample information and SH and I-HEDGE rankings from the network-based analyses.

Island	Species	*N*	SH	I-HEDGE
Pinta	*abingdoni*	12	4	1
Floreana	*nigra*	20	6	2
Santa Cruz	*donfaustoi*	20	1	3
San Cristóbal	*chathamensis*	19	3	4
Española	*hoodensis*	15	2	5
Pinzón	*ephippium*	27	5	6
Santa Cruz	*porteri*	23	7	7
Isabela	*microphyes*	21	8	8
Isabela	*vandenburghi*	28	9	9
Isabela	*becki*	45	10	10
Santiago	*darwini*	21	11	11
Isabela	*vicina*	116	12	12

**Notes.**

*N* sample size SH Shapley index I-HEDGE iterative heightened evolutionary distinctness globally endangered index

### Network construction

Pairwise differentiation among species was calculated with the fixation index *ϕ*_*ST*_ (Excoffier, Smouse & Quattro, 1992) using the Kimura 2-parameter (K2P) genetic distance and a gamma value of 0.5 (empirically-determined for CR sequences; Beheregaray et al., 2004), as implemented in Arlequin v3.5.1.2 (Excoffier, Laval & Schneider, 2005). The pair-wise differentiation matrix was then represented as a two-dimensional NeighbourNet network (Bryant & Moulton, 2004) using SplitsTree (Huson & Bryant, 2006) and default settings. This network representation produces sets of distances among subsets of taxa (termed “splits”) that can be used to calculate expected genetic contribution of individual tips (Volkmann et al., 2014).

### Prioritization metrics

As outlined by Volkmann et al. (2014), the expected future contribution of a taxon to total genetic diversity can be calculated by evaluating the split distance of a taxon to possible future subsets of taxa on a genetic network. The two metrics of future expected genetic diversity adapted to networks by Volkmann et al. (2014) are SH (Haake, Kashiwada & Su, 2008) and HED (Steel, Mimoto & Mooers, 2007). SH is based on game theory (Shapley, 1953), and calculates the predicted amount of diversity a taxon contributes to all possible subsets of taxa. HED is similar to SH, but weights each future subset of taxa based on its probability (Steel, Mimoto & Mooers, 2007). These probabilities are calculated by considering the probability of extinction (e.g., over the next 100 years) of each taxon in the network.

We used the scripts developed and published by Volkmann et al. (2014) to calculate SH, and modified the HED script to calculate I-HEDGE in the R statistical package (http://www.R-project.org/). HED values are used to calculate HEDGE, which is the product of HED and the p(ext) for the taxon. For the calculation of HED and HEDGE, it is important to use the best available information for the p(ext) of each taxon. When specific information is not available, it is possible to use proxies, such as those outlined in Mooers, Faith & Maddison (2008) that convert IUCN Red List (IUCN, 2014) statuses to p(ext) (e.g., Vulnerable = 0.1, Endangered = 0.667, Critically Endangered = 0.999). When this information is available, p(ext) can be informed by population viability analyses. The giant Galápagos tortoises are a special case where, despite being highly endangered, realistically they have a low actual p(ext) due to the intensive management they receive. In this case, the IUCN Red List statuses do not correlate to census population size, nor do they convert to a realistic probability of extinction for each species. We used a flat p(ext) for each of the extant taxa (arbitrarily set to 0.5) to reflect these circumstances. For the extinct species, p(ext) was set to 1 to reflect that, in fact, these species are extinct. I-HEDGE was calculated as follows. HEDGE was calculated initially for the entire set of taxa using p(ext) described above. The top-ranked taxon (eg., species X) was placed at the top of the I-HEDGE list. Next, assuming that species X will be “saved”, its extinction probability was then set to 0.001 and the HEDGE calculation was re-run. The top-ranked taxon from the second run that was not already prioritized was then given the overall second ranked position on the I-HEDGE list, its extinction probability was set to 0.001, and the procedure was repeated until all but one taxon has been prioritized. R scripts that automate the calculation of I-HEDGE from networks and trees can be found on GitHub (https://github.com/Eljensen/I-HEDGE).

The relationship between the species rankings from SH and I-HEDGE were then compared via simple Spearman’s rank correlation.

## Results

**Figure 2:**
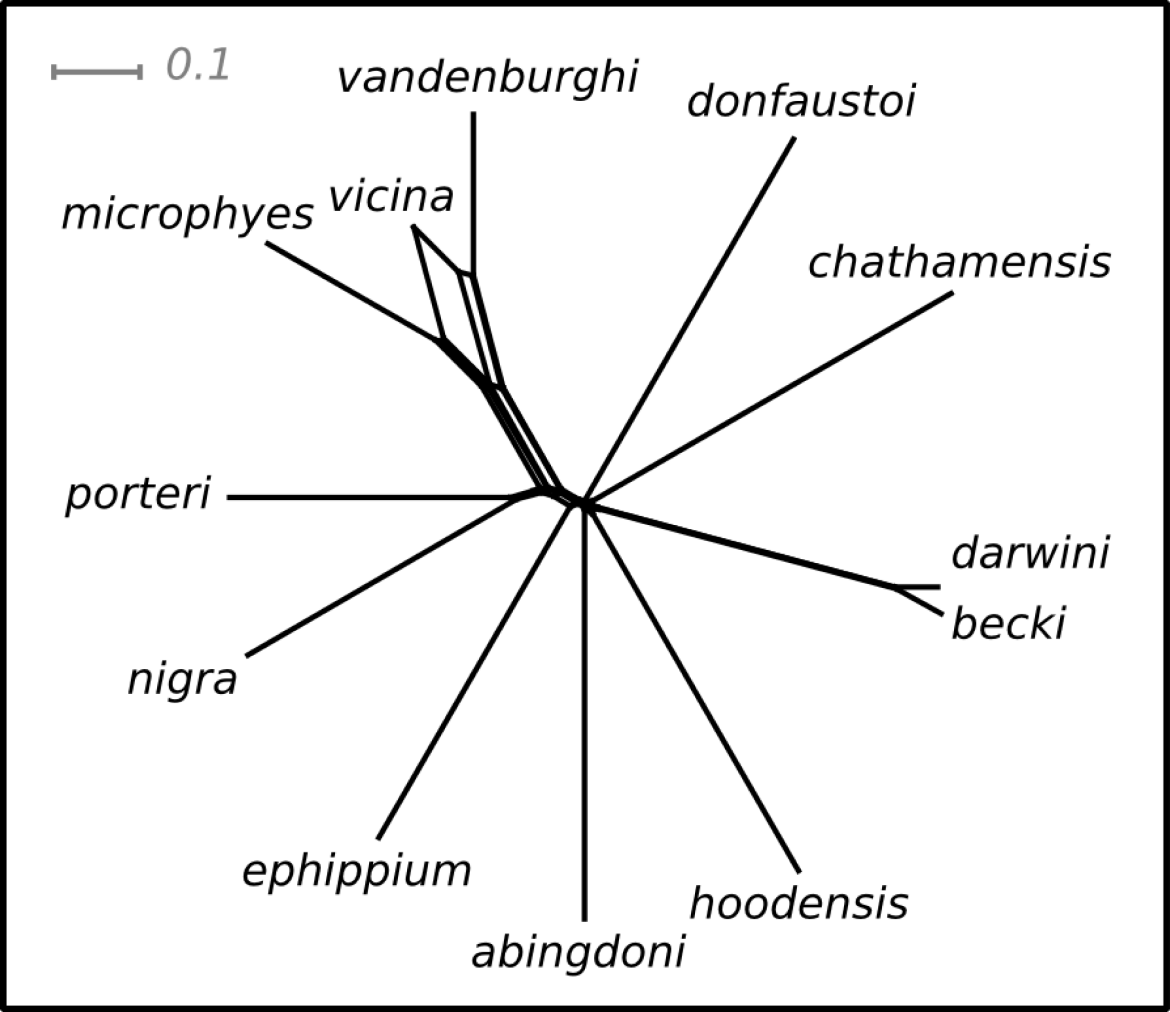
NeighbourNet depicting the relationships among species. The lengths of the edges on the network depict the degree of genetic differentiation.

Pairwise values of *ϕ*_ST_ ranged from 0.11 (*becki*—*darwini*) to 1 (*hoodensis*—*chathamensis*) among the species (Table S1). The network (Fig. 2) is non-treelike, and many of the terminals are roughly equally distant from the center of the network.

The ranking positions for the species estimated on this network were similar for both the SH and I-HEDGE metrics (Spearman’s rank correlation *ρ* = 0.8601, *p* < 0.0001). The ranking of the bottom seven species was identical between the metrics, while the top six varied by as many as four positions in the rankings (Table 1).

## Discussion

Often conservation decision makers are time and resource limited. The flexibility of input data for the I-HEDGE method is one of its strengths. The best available information regarding the p(ext) should be used, but in the absence of specific information, proxies can be used. Similarly, the network can be constructed from any type of differentiation matrix, including those generated from genotypic or phenotypic data. Furthermore, the network-based approach presented here can be applied below the species level to prioritize among populations or conservation units.

For the giant Galápagos tortoises, the SH and I-HEDGE ranking schemes produced similar results. Such robustness would be welcome, but the results are likely dependent on both network shape (here, quite starlike) and the patterns of imperilment across its tips. Here, we used a constant p(ext) for the extant species, but the results were also consistent using p(ext) values based on IUCN red lists statuses (data not shown) which vary from Vulnerable to Critically Endangered (Turtle Taxonomy Working Group et al., 2014). More simulation work and more case studies are needed to explore the sensitivity of these indices to variation in p(ext) and network/tree shape. Certainly, the iterative calculation of I-HEDGE should provide useful fine-tuning of the ranked list. The straight calculation of SH or HED values describes a property of the terminal unit, the average distance linking that unit to possible future networks. Such values should not be interpreted as an ordered list of priorities for conservation, since complementarity is not taken into account (Faith, 2008). In contrast, I-HEDGE produces a ranked list that can be used to identify the order of species that if conserved, would preserve the most future expected genetic diversity under a given set of extinction probabilities for tips. In the unlikely event of a tie, other factors could be taken into account (population size, logistics, available funding, etc.) to raise one taxon over the other. Indeed, we recognize that such other factors may take precedence over the priorities suggested by I-HEDGE. Nevertheless, by taking into account evolutionary isolation, probability of extinction and complementarity, I-HEDGE is an integrative index and provides a rational basis for conservation prioritization.

The greatest increase in phylogenetic diversity for the giant Galápagos tortoises can be achieved by restoring the two extinct species, *C. abingdoni* and *C. nigra*. This result is due to the fact that they currently contribute no phylogenetic diversity and, if restored, would each contribute an edge of substantial length on the network. Evaluating the contributions of these species to overall diversity is timely, as they may not be extinct for much longer: individuals with admixed ancestry have been discovered that share as much as half their genomes with these recently extinct species from Floreana Island (Poulakakis et al., 2008; Russello et al., 2010; Garrick et al., 2012) or Pinta Island (Russello et al., 2007; Edwards et al., 2013). The Galápagos National Park has begun an initiative to retrieve these admixed individuals and use them for both selective breeding and repatriation to their respective islands. Our finding that the greatest increase in phylogenetic diversity can be achieved by resurrecting the two extinct species provides additional support to the initiative.

Over the last 50 years, the species that have received the most intensive management are *C. hoodensis*, which was rescued from a population low of 15 individuals to its current size numbering nearly 2000 through captive breeding (Milinkovitch et al., 2013; Gibbs et al., 2014) and *C. ephippium*, which was the focus of a head-start program (Cayot, 2008). Our finding that these species rank fifth and sixth, respectively, for I-HEDGE further substantiates the extreme efforts that were put into recovering them from the brink of extinction.

As the shape of the network directly impacts the ranking of the terminal units, it is important to use genetic markers that are appropriate to the scale of divergence among taxa and reflect genome wide genetic diversity. Here, we made use of an existing, expansive mitochondrial control region dataset that has proven informative across multiple studies at both the within- and among-population/species levels in giant Galápagos tortoises (Caccone et al., 2002; Russello et al., 2005; Russello et al., 2007; Poulakakis et al., 2008; Garrick et al., 2012; Poulakakis et al., 2012; Edwards et al., 2013). We evaluated a previously published microsatellite dataset for giant Galápagos tortoises (Garrick et al., 2015) for use in this study, but the network generated depicted relationships that were highly incongruent with all previous studies of this group based on nuclear and mitochondrial DNA character data (see Fig. S1) (Caccone et al., 2004; Poulakakis et al., 2012). Homoplasy of microsatellite fragment lengths has never been investigated in giant Galápagos tortoises, but studies of other taxa have found this to be quite common in comparisons among recently diverged groups (Garza & Freimer, 1996; Angers, Estoup & Jarne, 2000; Van Oppen et al., 2000; Anmarkrud et al., 2008). Given the wide range of divergence times between giant Galápagos tortoises (<0.28 mya–1.7 mya; Poulakakis et al., 2012), it is quite likely that this source of homoplasy may have contributed to the reconstruction of spurious relationships that would influence downstream rankings. We therefore decided that the microsatellite data was not appropriate to use in this context, and suggest that marker choice should be given careful consideration on a system-by-system basis prior to implementing this network-based approach. For example, Volkmann et al. (2014) used two case studies to initially illustrate the calculation of SH and HED from networks, one using mitochondrial control region data for a broadly distributed species with subspecific variation, and another finer-scale example using microsatellite genotypic data for an endemic species with a highly restricted distribution. We recognize that basing conservation priorities on the information in a single locus is not ideal, and moving forward, genome-wide single nucleotide polymorphism data may be best suited to this approach, providing broad-scale coverage that enables more precise estimation of population-level parameters, including structure within and among populations and species.

## Conclusions

The giant Galápagos tortoises are among the most charismatic emblems of evolutionary biology, and flagship species for conservation. Our results support both past and ongoing recovery efforts, and reinforce the emphasis that has been placed on rescuing *C. ephippium* and *C. hoodensis* from the brink of extinction over the past 50 years. The possible revival of two recently extinct species *C. abingdoni* and *C. nigra*, if successful, may contribute substantially to the total genetic diversity of the giant Galápagos tortoises. As the Anthropocene progresses, it is important that conservation decisions are deliberate and based on the best available information. Metrics that explicitly measure a taxon’s expected genetic contributions to future biodiversity, especially those that incorporate complementarity (such as I-HEDGE, introduced here) may be useful tools for managers interested in stewarding the breadth of genetic diversity under the Noah’s Ark paradigm. As a general prioritization program moves forward, it will be important to identify both the axes of worth (ecological, evolutionary, current utility), and, for each, identify appropriate metrics (e.g., reliable measures of genetic diversity).

##  Supplemental Information

10.7717/peerj.2350/supp-1 Table S1 Pairwise values of *φ*_ST_ among species Pairwise values of *f*_ST_ among species, calculated using the Kimura 2-parameter model. Each value is significant at *p* < 0.001, except between *darwini* and *becki*, which is significant at *p* < 0.05.

10.7717/peerj.2350/supp-2 Figure S1 Depictions of the relationships among Galápagos tortoise species from previous studies in comparison to the splits networks generated from control region and microsatellite datasets Depictions of (A) the relationships among Galápagos tortoise species resolved by previous studies (Beheregaray et al., 2004; Caccone et al., 2002; Russello et al., 2005; Poulakakis et al., 2008; Poulakakis et al., 2012) which are here presented as an unrooted equal-length tree, (B) the splits-network generated from pairwise *φ*_ST_ values calculated from mitochondrial control region sequences and (C) the splits-network generated from pairwise *D*_est_ (Jost 2008) values calculated from genotypes at 12 microsatellite loci. While A and B represent similar patterns, C depicts divergent relationships, particularly the placement of *hoodensis* away from *abingdoni*, *chathamensis* away from *donfaustoi*, and *nigra* away from *porteri*.

10.7717/peerj.2350/supp-3 Supplemental Information 1 R script to calculate I-HEDGE from a network

10.7717/peerj.2350/supp-4 Supplemental Information 2 R script to calculate I-HEDGE from a tree

## Additional Information and Declarations

### Data Availability

The following information was supplied regarding data availability: The research in this article did not generate any raw data. We made use of the following database (DRYAD entry doi:10.5061/dryad.7h8q2).
