# Supplementary material for: I-HEDGE: determining the optimum complementary sets of taxa for conservation using evolutionary isolation
Source: PeerJ. 2016 Aug 23;4:e2350. doi: 10.7717/peerj.2350 (PMC5012326; doi:10.7717/peerj.2350)
Supplement: Table S1 — Pairwise values of fST among species, calculated using the Kimura 2-parameter model. Each value is significant at p < 0.001, except between darwini and becki, which is significant at p < 0.05. [file peerj-04-2350-s001.pdf]

I-HEDGE: Determining the optimum complementary sets of taxa for conservation using evolutionary isolation

Evelyn L. Jensen, Arne Ø. Mooers, Adalgisa Caccone, and Michael A. Russello

Table S1. Pairwise values of ϕ_{ST} among species, calculated using the Kimura 2-parameter model.

Each value is significant at p <0.001, except between *darwini* and *becki*, which is significant at p <0.05.

	darwini	*donfaustoi*	*hoodensis*	*porteri*	*becki*	*abingdoni*	*ephippium*	*chathamensis*	*vandenburghi*	*microphyes*	*nigra*	*vicina*
darwini	0.00											
donfaustoi	0.92	0.00										
hoodensis	0.91	1.00	0.00									
porteri	0.84	0.91	0.93	0.00								
becki	0.11	0.92	0.92	0.87	0.00							
abingdoni	0.92	0.98	0.98	0.91	0.93	0.00						
ephippium	0.90	0.96	0.96	0.84	0.91	0.95	0.00					
chathamensis	0.91	0.99	1.00	0.92	0.92	0.98	0.96	0.00				
vandenburghi	0.93	0.99	0.99	0.85	0.93	0.98	0.95	0.99	0.00			
microphyes	0.92	0.99	0.99	0.81	0.92	0.98	0.94	0.99	0.67	0.00		
nigra	0.86	0.94	0.96	0.70	0.88	0.94	0.89	0.95	0.90	0.88	0.00	
vicina	0.85	0.88	0.89	0.74	0.87	0.89	0.85	0.89	0.27	0.36	0.71	0.00
