# Supplementary material for: I-HEDGE: determining the optimum complementary sets of taxa for conservation using evolutionary isolation
Source: PeerJ. 2016 Aug 23;4:e2350. doi: 10.7717/peerj.2350 (PMC5012326; doi:10.7717/peerj.2350)
Supplement: Figure S1 — Depictions of (A) the relationships among Galápagos tortoise species resolved by previous studies (Beheregaray et al., 2004; Caccone et al., 2002; Russello et al., 2005; Poulakakis et al., 2008; Poulakakis et al., 2012) which are here presented as an unrooted equal-length tree, (B) the splits-network generated from pairwise φST values calculated from mitochondrial control region sequences and (C) the splits-network generated from pairwise Dest (Jost 2008) values calculated from genotypes at 12 microsatellite loci. While A and B represent similar patterns, C depicts divergent relationships, particularly the placement of hoodensis away from abingdoni, chathamensis away from donfaustoi, and nigra away from porteri. [file peerj-04-2350-s002.pdf]

I-HEDGE: Determining the optimum complementary sets of taxa for conservation using evolutionary isolation

Evelyn L. Jensen, Arne Ø. Mooers, Adalgisa Caccone, and Michael A. Russello

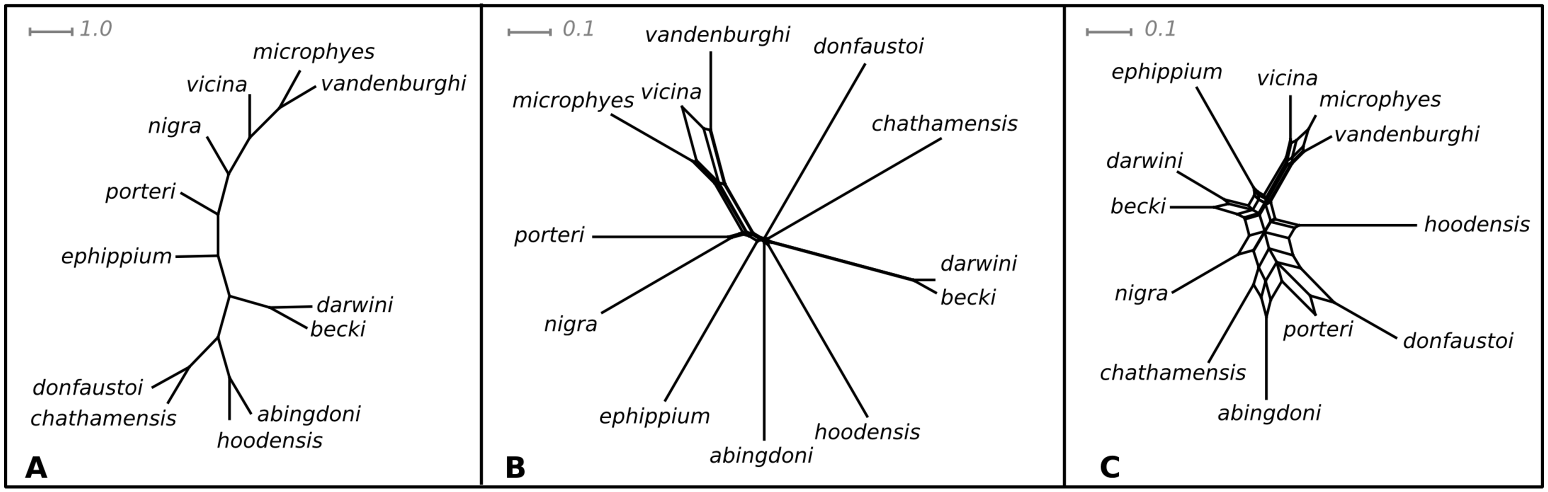

Figure S1

Depictions of (A) the relationships among Galápagos tortoise species resolved by previous studies (Beheregaray et al. 2004; Caccone et al. 2004; Russello et al. 2005; Poulakakis et al. 2008; Poulakakis et al. 2012) which are here presented as an unrooted equal-length tree, (B) the splits-network generated from pairwise ϕ_{ST} values calculated from mitochondrial control region sequences and (C) the splits-network generated from pairwise D_{est} (Jost 2008) values calculated from genotypes at 12 microsatellite loci. While A and B represent similar patterns, C depicts divergent relationships, particularly the placement of *hoodensis* away from *abingdoni*, *chathamensis* away from *donfaustoi*, and *nigra* away from *porteri*.

Supplementary References:

Beheregaray LB, Gibbs JP, Havill N, Fritts TH, Powell JR, Caccone A (2004) Giant tortoises are not so slow: rapid diversification and biogeographic consensus in the Galápagos. Proc Natl Acad Sci U S A 101:6514-6519. doi:10.1073/pnas.0400393101

Caccone A, Gentile G, Burns CE, Sezzi E, Bergman W, Ruelle M, Saltonstall K, Powell JR (2004) Extreme difference in rate of mitochondrial and nuclear DNA evolution in a large ectotherm, Galapagos tortoises. Mol Phylogenet Evol 31:794-798. doi:10.1016/j.ympev.2004.02.004

Jost L (2008) GST and its relatives do not measure differentiation. Mol Ecol 17:4015-4026. doi:10.1111/j.1365-294X.2008.03887.x

Poulakakis N, Glaberman S, Russello M, Beheregaray LB, Ciofi C, Powell JR, Caccone A (2008) Historical DNA analysis reveals living descendants of an extinct species of Galápagos tortoise. Proc Natl Acad Sci U S A 105:15464-15469. doi:10.1073/pnas.0805340105

Poulakakis N, Russello M, Geist D, Caccone A (2012) Unravelling the peculiarities of island life: vicariance, dispersal and the diversification of the extinct and extant giant Galápagos tortoises. Mol Ecol 21:160-173. doi:10.1111/j.1365-294X.2011.05370.x

Russello MA, Glaberman S, Gibbs JP, Marquez C, Powell JR, Caccone A (2005) A cryptic taxon of Galápagos tortoise in conservation peril. Biol Lett 1:287-290. doi:10.1098/rsbl.2005.0317
